# Overexpression of leucoanthocyanidin reductase or anthocyanidin reductase elevates tannins content and confers cassava resistance to two-spotted spider mite

**DOI:** 10.3389/fpls.2022.994866

**Published:** 2022-08-18

**Authors:** Qing Chen, Xiao Liang, Chunling Wu, Ying Liu, Xiaoqiang Liu, Huiping Zhao, Kaimian Li, Songbi Chen, Haiyan Wang, Zhiling Han, Mufeng Wu, Xiaowen Yao, Jun Shui, Yang Qiao, Xue Zhan, Yao Zhang

**Affiliations:** ^1^Environment and Plant Protection Institute, Chinese Academy of Tropical Agricultural Sciences, Key Laboratory of Integrated Pest Management on Tropical Crops, Ministry of Agriculture and Rural Affairs, Haikou, Hainan, China; ^2^Sanya Research Academy, Chinese Academy of Tropical Agriculture Science, Hainan Key Laboratory for Biosafety Monitoring and Molecular Breeding in Off-Season Reproduction Regions, Sanya, Hainan, China; ^3^Tropical Crops Genetic Resources Institute, Chinese Academy of Tropical Agriculture Sciences, Haikou, China; ^4^Institute of Tropical Bioscience and Biotechnology, Chinese Academy of Tropical, Haikou, China

**Keywords:** cassava (*Manihot esculenta* Crantz), two-spotted spider mite (*Tetranychus urticae* Koch), leucoanthocyanidin reductase, anthocyanidin reductase, tannins (condensed), resistance mechanism

## Abstract

The two-spotted spider mite (TSSM) is a destructive cassava pest. Intensive demonstration of resistance mechanism greatly facilitates the creation of TSSM-resistant cassava germplasm. Gene to metabolite network plays a crucial role in modulating plant resistance, but little is known about the genes and related metabolites which are responsible for cassava resistance to TSSM. Here, a highly resistant (HR) and a highly susceptible (HS) cassava cultivar were used, integrative and comparative transcriptomic and metabolomic analyses between these two cultivars after TSSM infestation revealed that several genes and metabolites were closely related and significantly different in abundance. In particular, the expression of leucoanthocyanidin reductase (*LAR*) and anthocyanidin reductase (*ANR*) genes showed a high positive correlation with most of the metabolites in the tannin biosynthesis pathway. Furthermore, transgenic cassava lines overexpressing either of the genes elevated tannin concentrations and conferred cassava resistance to TSSM. Additionally, different forms of tannins possessed distinct bioactivity on TSSM, of which total condensed tannins (LC_50_ = 375.68 mg/l) showed maximum lethal effects followed by procyanidin B1 (LC_50_ = 3537.10 mg/l). This study accurately targets *LAR*, *ANR* and specific tannin compounds as critical genes and metabolites in shaping cassava resistance to TSSM, which could be considered as biomarkers for evaluation and creation of pest-resistant cassava germplasm.

## Introduction

Cassava (*Manihot esculenta* Crantz) is an ancient root and tuber crop widely cultivated in over 100 countries in the tropical and subtropical regions of Africa, America, and Asia (Parmar et al., 2017). This crop not only constitutes the staple food for more than 900 million people in developing countries, but also is used as animal feed and for bio-industrial applications worldwide (Wu et al., 2022). The two-spotted spider mite (TSSM; *Tetranychus urticae;* Acari: Tetranychidae), the most polyphagous species of the Tetranychidae family, is a serious threat to several crops worldwide, including cassava. In China, this mite has been shown to cause about 50–70%, sometimes even 100% yield loss (Chen et al., 2019). Although using resistant cassava cultivars is considered an eco-friendly, effective, and economical strategy for controlling cassava insect pests (Bellotti et al., 2012), the predominant management practices to control TSSM are still largely reliant on pesticides. However, the inappropriate application of pesticides may also kill natural enemies and lead to potential resistance problems (Leeuwen et al., 2008). Furthermore, despite considerable efforts to evaluate and screen cassava genotypes to identify insect pest resistance, few resistant commercial varieties have been released for cultivation over the past decades (Bellotti et al., 2012). In addition, limited efforts have been made to decipher the mechanisms of cassava resistance to mites.

Plants possess both indirect and direct defense mechanisms to cope with insect pest infestation. Indirect defense involves the emission of herbivore-induced plant volatiles that attract natural enemies to reduce pest density and plant damage (Aljbory and Chen, 2016). In direct defense, in addition to physical barriers (i.e., thickness and trichomes), plants mobilize a variety of defense signaling pathways, such as jasmonic acid (JA), salicylic acid (SA), ethylene (ET), abscisic acid (ABA), brassinosteroids (BR), and gibberellic acid (GA) pathways, and activate the expression of defense-related genes responsible for the synthesis of secondary metabolites (Jin et al., 2016). The secondary metabolites, such as antifeedants or toxic compounds that inhibit insect pest performance (Howe and Jander, 2008), have been recognized as critical factors shaping plant resistance to insect pests (Hu et al., 2019). The synthesis of specific secondary metabolites is regulated by a battery of specific genes (Mugford et al., 2013). Therefore, building up a gene-metabolite network could reveal the resistance mechanism or mine candidate genes for further molecular breeding of resistant cultivars (Ma et al., 2019), which requires an integrated analysis based on multiple genes and closely related metabolite levels. Furthermore, several studies have speculated an integrated investigation of the TSSM resistance mechanism and the mining of vigorous resistant phenotypes, traits, or genes in cassava can assist the identification and breeding of TSSM-resistant cassava cultivars (Agut et al., 2018).

Numerous studies have used high-throughput technologies (i.e., genomics, transcriptomic, proteomic, and metabolomic analyses) to investigate the regulation of plant resistance to insect pests. For instance, a study employing transcriptomic analysis demonstrated that during Arabidopsis–TSSM interaction the indole-glucosinolates (IG) are induced by TSSM feeding, in addition, the accumulation of IG significantly increases the mortality of TSSM, furthermore, a positive regulator of the IG gene, *Altered tryptophan regulation 1* (*ATR1*), was identified using transcriptomic analysis, and the overexpression of *ATR1* increased the resistance of plants to spider mite infestation and larval mortality (Zhurov et al., 2014). In addition, several studies using either mono-omics or multi-omics analyses have provided molecular insights into the gene-metabolite regulation network involved in resistance of the plants to TSSM in several crops, including tomato (Weinblum et al., 2021), cucumber (He et al., 2020), pepper (Zhang et al., 2019), bean (Scott et al., 2021), barley (Santamaria et al., 2018) and citrus (Agut et al., 2014). In recent years, transcriptomic and proteomic analyses have been used to unravel the resistance mechanism of cassava to TSSM and its related species (*T. cinnabarinus*; Yang et al., 2019, 2020). Furthermore, accumulating studies have demonstrated the potential of multi-omics integrative analysis as a promising tool for examining complex physiological processes (Ghag et al., 2015; Hu et al., 2019) and identifying the genes, proteins, and metabolites responsible for the resistance of plants to TSSM (Agut et al., 2018). These techniques have also been used to depict the potential resistance regulatory networks. In the studies described above, a large class of metabolites, such as flavonoids (Yang et al., 2019, 2020; Scott et al., 2021), phenylpropanoids (Yang et al., 2019), terpenes (Zhang et al., 2019), cucurbitacins (He et al., 2020) and cystatins (Santamaria et al., 2018), are thought to be responsible for TSSM resistance, among which the phenylpropanoid and flavonoid pathways were frequently found to contribute to insect pest resistance (Yang et al., 2020). However, as these metabolite families contain hundreds of chemicals, to specify which chemicals prominently contribute to plant resistance could be quite tricky. Moreover, there is a lack of further experimental validation steps to confirm the specific genes and related metabolites involved in shaping plant resistance to TSSM.

Therefore, we hypothesized that a few rather than a battery of genes or toxic compounds might play predominant role in conferring cassava resistance to mite, in addition, understanding the different responses between resistant and susceptible plants can help in deciphering the mite-resistance mechanisms. To test this hypothesis in present study, a highly resistant (HR) and a highly susceptible (HS) cassava cultivar that identified in previous study were used (Liang et al., 2017), and numerous genes and metabolites that may contribute to TSSM resistance were identified, in addition, the function of certain important resistant related genes and metabolites were validated using either gene transformation method or *in vitro* bioassay. This study could provide insights into potential genes and the mechanism of TSSM resistance and assist in the molecular breeding of pest-resistant cassava.

## Materials and methods

### Cultivation of cassava plants

The highly resistant (HR) cassava cultivar C1115 and the highly susceptible (HS) cassava cultivar BRA900 that were identified in our previous study (Liang et al., 2017; Lu et al., 2017) were supplied by the National Cassava Germplasm Nursery of China, Chinese Academy of Tropical Agricultural Sciences (CATAS). Cassava stem segments with at least three eyes were vertically planted in pots (33 cm diameter, 25 cm height) containing 5 kg of well-mixed soil (soil: peat: perlite = 1:1:1) and grown in a greenhouse [L14:D10 (light/dark) photoperiod].

### Laboratory rearing of *Tetranychus urticae*

*Tetranychus urticae* rearing was performed following a previously described method (Liang et al., 2017). Healthy adults were maintained by the Environment and Plant Protection Institute, CATAS, and reared on the backs of healthy cassava leaves of BRA900 cultivars at 28 ± 1°C, 75 ± 5% relative humidity, and L14:D10 photoperiod. A water-saturated blotting paper strip was wrapped around the leaf margin to prevent the escape of mites and to keep the leaves fresh. The leaves were replaced every 2–3 days.

### *Tetranychus urticae* infection treatment and sample collection

Cassava leaves of identical growth from the middle of the 3-month-old laboratory plants were selected, 50 healthy female adult mites were inoculated on the back of a whole leaf of either highly resistant (HR) cultivar or the highly susceptible (HS) cassava cultivar., and petioles were coated with vaseline to prevent the escape of mites. Leaves before mite infestation (0 day without infestation, HR0 and HS0), short-term mite infestation (infested by TSSM for 1 day, HR1 and HS1) and long-term mite infestation (infested by TSSM for 8 day, HR8 and HS8) were sampled. There were three replicates per treatment, with one infested plant representing a replicate. We set these time points for sampling were based on our previous study, in which distinct differences in both physiological and biochemical responses were observed (Liang et al., 2017; Lu et al., 2017).

### Transcriptome analysis

RNA sequencing (RNA-seq) and library construction were performed by Novogene Bioinformatics Technology Co. Ltd. (Tianjin, China). Briefly, RNA (~ 3 μg) from each sample was extracted using the RNApre Pure Plant Plus Kit (TIANGEN, Beijing, China) according to the manufacturer’s instructions. Samples from three biological replicates were used in this study. Sequence quality was examined using the FastQC software.^1^ Clean reads were aligned to the cassava reference genome (version 6.1) obtained from the Phytozome database^2^ using TopHat v2.0.10 (Kim et al., 2013). Differentially expressed genes (DEGs) were determined using DESeq2 (Love et al., 2014) with log2|fold-change| > 1 and a false discovery rate of < 0.05. Hierarchical clustering was performed using pheatmap v.1.0.12 R package. Kyoto Encyclopedia of Genes and Genomes (KEGG) enrichment analyses of the DEGs were performed using the cluster Profiler R package.

### Metabolome analysis

Approximately 200 mg of freeze-dried leaves was homogenized in liquid nitrogen and subjected to non-targeted metabolomics analysis (Metware Biotechnology Co., Ltd., Wuhan, China). Samples from three biological replicates were used in this study. The sample extracts were analyzed using a UPLC-ESI-MS/MS system (Shim-pack UFLC SHIMADZU CBM30A system; MS, Applied Biosystems 4,500 Q TRAP) following the analysis conditions described in a previous study (Ding et al., 2020). Metabolites were identified using information from public metabolite databases and the Metware database (Metware Biotechnology Co., Ltd. Wuhan, China). All identified metabolites were subjected to principal component analysis (PCA), and significant differences were determined by setting the variable importance in projection (VIP) to ≥ 1 and log2 |fold-change| ≥ 1. Hierarchical clustering was performed using pheatmap. KEGG enrichment analyses of the differentially expressed metabolites (DEMs) were performed using the Cluster Profiler.

### Integrated transcriptome and metabolome analysis

The transcripts and metabolite abundances were min-max normalized between −1 and 1 at different time points (Becker et al., 2018). Abundance patterns were determined using the standard procedure of the WGCNA R package (Langfelder and Horvath, 2008) and subsequently visualized using pheatmap. Transcripts and metabolites not assigned to any group were excluded from further analysis.

To interpret the biological functions of DEGs and DEMs, cassava genes were functionally annotated and assigned to different hierarchical categories using the MapMan classification system (Thimm et al., 2004). The significance of the enriched categories was determined using Fisher’s exact test. Pearson’s correlation test was used to calculate the correlation coefficients between DEGs and their corresponding metabolites. WGCNA was used to determine the association between mRNAs and metabolites.

### RT-qPCR

RT-qPCR was performed on a Roche LightCycler96 Real-Time PCR System (Roche Diagnostics Ltd., Rotkreuz, Switzerland). The PCR reactions consisted of 7 μl of double-distilled water (ddH_2_O), 10 μl of 2 × SYBR^®^ Premix Ex TaqTM (TaKaRa, Shiga, Japan), 0.5 μl of each specific forward and reverse primer, and 2 μl of first-strand cDNA (5-fold diluted cDNA) in a final volume of 20 μl. The RT-qPCR conditions were: an initial denaturation for 2 min at 95°C, followed by 40 cycles of denaturation at 95°C for 5 s and annealing at 60°C for 30 s, and a final elongation step at 72°C for 60 s. For the melting curve analysis, a dissociation step cycle (65°C for 5 s, and then an increase of 0.5°C every 10 s up to 95°C) was used. The reactions were performed in 96-well PCR plates in triplicate (technical replicates) for each biological sample. Cassava actin (KM583807.1) was used as a reference, and the relative quantification was calculated based on the comparative 2^–ΔΔCt^ method (Livak and Schmittgen, 2001). The primer information is shown in Supplementary Table S1.

### Activity analyses of tannins synthesis-related enzymes

The activities of tannin-synthesis-related enzymes, such as flavanone 3-hydroxylase (F3H), dihydro flavonol reductase (DFR), anthocyanidin synthase (ANS), leucoanthocyanidin reductase (LAR), and anthocyanidin reductase (ANR), were analyzed using ELISA kits (Shanghai Enzyme-linked Biotechnology Co., Ltd., Shanghai, China) according to the manufacturer’s instructions for the corresponding kits. Each treatment was replicated at least thrice.

### Plasmid construction and transformation of tannins synthesis gene *MeLAR* or *MeANR* in cassava

The coding sequences (CDS) of *MeLAR* (accession no. OAY31259.1) and *MeANR* (accession no. OAY39177.1) were PCR-amplified using cassava cDNA (from the HR cultivar C1115) and sequenced. The *MeLAR* and *MeANR* fragments were inserted individually into the pCAMBIA1301S plant expression vector containing the CaMV35S promoter to generate the pC-35S::*MeLAR* and pC-35S::*MeANR* vectors, respectively.

*Agrobacterium tumefaciens* strain LBA4404, harboring the above vectors, was used for cassava transformation. Shoot meristems from the apical and axillary buds of the cassava cultivar TMS60444 were used as wild type (WT) explants for embryogenic callus induction, TMS60444 was one of the mature plant materials for cassava transformation (Xu et al., 2013; An et al., 2017) and was identified as mite-susceptible cultivars in our previous study (Lu et al., 2017). Propagation of embryogenic calli, transformation with *A. tumefaciens*, and plant regeneration from embryonic calli were performed following the method described previously (Xu et al., 2013). Putative transgenic plants were rooted in BBM medium (SBM supplemented with 10 mg/l hygromycin) and subcultured. Molecular and phenotypic characterizations of the wild type (WT) and transgenic cassava lines were performed using Southern blotting and PCR analysis (Primer sequences can be seen in Supplementary Table S1), respectively. Southern blot analysis was conducted following a previously described method (Xu et al., 2013) to confirm the stable integration of the transgenes into the nuclear genome of the regenerated cassava plants. In addition, qPCR was used to analyze the expression of *MeLAR* and *MeANR*, respectively.

### Determination of terminal and extension units of condensed tannins

The tannin content was optimized by precipitation methods, as described by Benahmed (Benahmed Djilali et al., 2021). Tannin yield was calculated as the following formula:

Tannin yield % = (*W*1/*W*0) × 100%, where *W*1: weight of dried extracted tannins, *W*0: weight of sample used.

The tannin content in the extract was determined using the casein colorimetric method (Benahmed Djilali et al., 2021). Casein (1 g) was added to 6 ml of sample extract, followed by the addition of 12 ml distilled water. The solution was then mixed for 3 h on a shaker at 150 rpm. The mixture was then filtered, and the filtrate was made up to 25 ml using distilled water. Tannin content was calculated as the difference between the total flavonoid content and the total flavonoid content obtained after tannin complexation by casein (Sigma, United States). The hydrolysis of extracted condensed tannins from cassava leaf sample and the determination of its terminal and extension units were conducted as described by Zhang and Lin (2008) using reversed-phase HPLC with minor modification. An Agilent 1,100 system (United States) equipped with a diode array detector and a quaternary pump was used. A Hypersil ODS column (4.6 mm × 250 mm, 2.5 μm) was used. 0.1% aqueous trifluoroacetic acid and acetonitrile was set as mobile phase. The elution system was: 0–5 min, 5% B (isocratic); 5–15 min, 5–10% B (linear gradient); 15–45 min, 10–15% B (linear gradient), 45–60 min, 16–60% B (linear gradient). The column temperature was ambient and the flow-rate was set at 1 ml/min. Detection was at 280 nm and the UV spectra were acquired between 200 and 600 nm. Degradation products were identified on chromatograms according to their retention times and UV-visible spectra. Peaks were manually integrated, and quantification was performed by reporting the measured area into the calibration curve of the corresponding compound. Once the terminal and extension units of condensed tannins were confirmed, their contents in the cassava leaf samples (before and after infestation by TSSM) were further analyzed.

### Performance of transgenic cassava lines against TSSM infestation

Transgenic cassava lines overexpressing *MeLAR* or *MeANR* genes were subjected to TSSM infestation. At 1 and 8 day-post-infestation, the transcription and enzyme activity of LAR and ANR, and the concentrations of condensed tannins and their terminal and extension units were analyzed. In addition, the influence of cassava feeding on mortality, hatchability, and development duration of TSSM using the transgenic, WT, HS, and HR cassava plants was recorded. Moreover, the TSSM-resistance level of transgenic cassava in the field was evaluated based on the mite damage index method that developed in our previous study (Lu et al., 2017), in which the resistant level and corresponding mite damage index were listed as: immunity (0.0%), highly resistant (0.1–12.5%), resistant (12.6–37.5%), moderate resistant (37.6–62.5%), susceptible (62.5–87.5%)and highly susceptible (>87.5%).

### Bioassay of condensed tannins and their terminal and extension units on TSSM

Treatments with the tannin compounds were performed using the soaking method. As high concentrations of these compounds are not completely soluble in water, they were first dissolved in acetone and then diluted with 5% acetone-ddH_2_O to a series of concentrations (50, 100, 200, 1,600, 3,200, and 6,400 mg/l). The mites were soaked in these solutions for 20 min. After soaking, the mites were gently washed with ddH_2_O and transferred onto filter paper for drying. Once the mites were dry, they recovered and started moving. The recovered adults were transferred onto the BRA900 leaf with a micropipette and continued to be reared under the conditions described in the previous section (Laboratory rearing of *T. urticae*). Each treatment was replicated at least three times.

### Statistical analyses

All data obtained were processed in SPSS, and statistical analysis was conducted using one-way analysis of variance (ANOVA) with Tukey’s honestly significant difference (HSD) multiple comparison test. Significant and extreme differences were considered if *p*-values were < 0.05 and < 0.01, respectively.

## Results

### Transcriptomic analysis revealed important genes involved in chemical defensive responses to TSSM infestation

**Figure 1:**
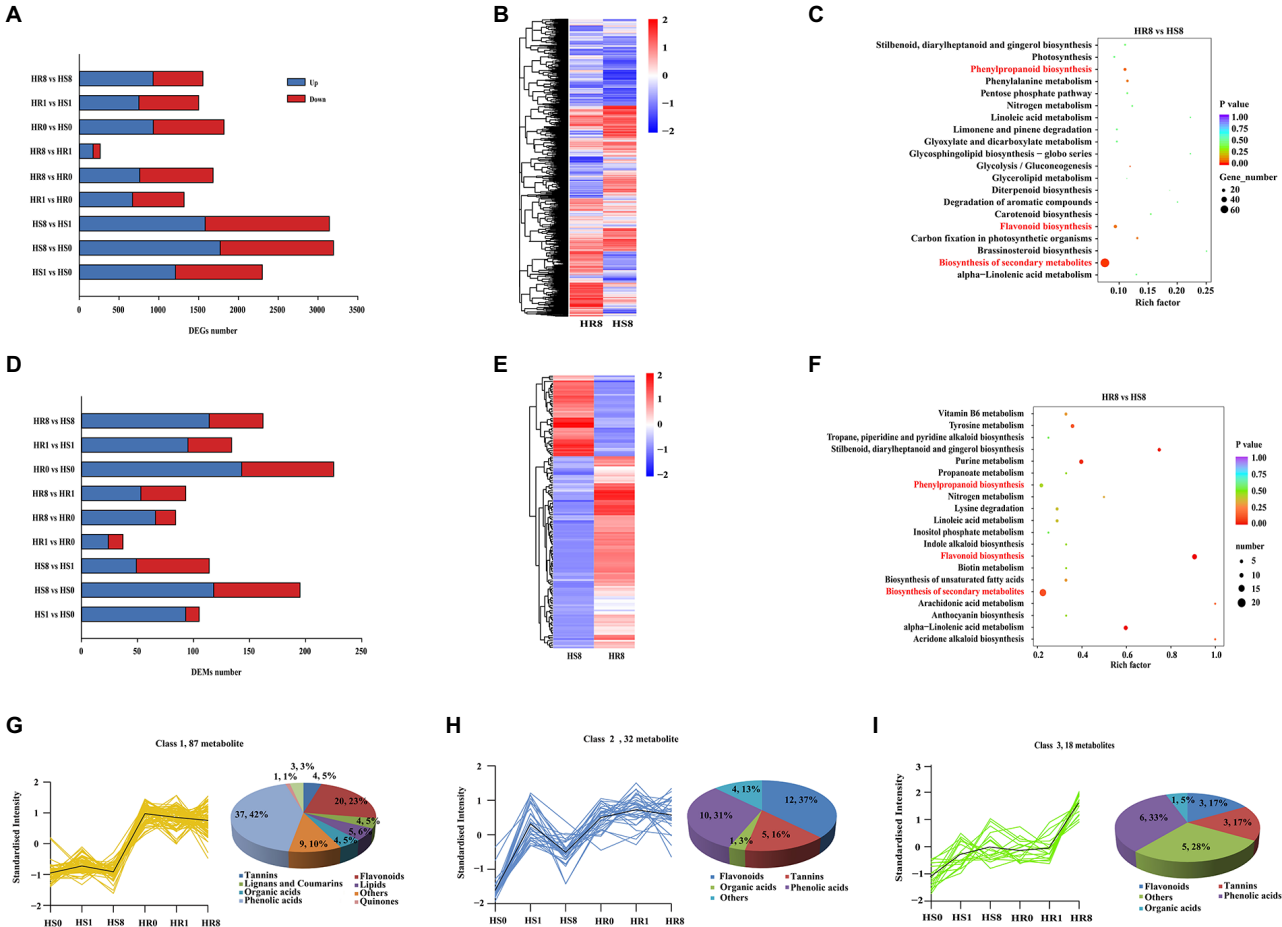
Comparative transcriptome and metabolome analysis of HR and HS cassava cultivars under TSSM infestation. **(A)** The overview of differently expressed genes (DEGs) between HR and HS cultivars at different infestation time points [0 d (HR0 and HS0), 1 d (HR1 and HS1), and 8 d (HR8 and HS8)]. **(B)** Heatmap cluster analysis of DEGs in the “HR8 vs. HS8” group. **(C)** The Kyoto Encyclopedia of Genes and Genomes (KEGG) enrichment analysis of DEGs in the “HR8 vs. HS8” group, the pathways highlighted with red font indicate either abundant genes or high rich factors. **(D)** The overview of differently expressed metabolites (DEMs) between HR and HS cultivars at different infestation time points (0, 1, and 8 d). **(E)** Heatmap cluster analysis of DEMs in the “HR8 vs. HS8” group. **(F)** The KEGG enrichment analysis of DEMs in the “HR8 vs. HS8” group. The pathways highlighted with red font indicate either abundant genes or high rich factors. **(G–I)** The representative DEMs classes enriched with most flavonoids and tannins compounds in different samples.

To gain an overview of the gene transcriptions induced by TSSM, transcriptomic analyses of cassava leaf samples was performed (see Sequence Read Archive database, accession ID: PRJNA822050). Overall, ~ 443 million paired-end reads were obtained by Illumina sequencing, with an average of 24.8 million reads per sample library. Principal component analysis of the transcriptome data of C1115 and BRA900 at different time points of infestation identified distinct clusters for the HR and HS groups (Supplementary Figure S1A). Comparison of the DEGs between the two groups revealed more abundant DEGs in the HS group (i.e., HS1 vs. HS0, HS8 vs. HS0 and HS8 vs. HS1) than in the HR group (i.e., HR1 vs. HR0, HR8 vs. HR0 and HR8 vs. HR1; Figure 1A). The DEGs between HR8 and HR1 samples consisted only 174 upregulated and 89 downregulated genes, while the pair-comparison groups HR0 vs. HR1 (669 upregulated and 648 downregulated genes) and HR0 vs. HR8 (759 upregulated and 923downregulated genes) identified more DEGs, suggesting a distinct different response before and post-infestation by TSSM (Figure 1A). The heat map analysis identified distinct clusters of the DEGs between the HR and HS groups (HS0 vs. HR0, HS1 vs. HR1, and HS8 vs. HR8; Figure 1B; Supplementary Figures S2A,B). Furthermore, KEGG enrichment analyses identified that “biosynthesis of secondary metabolite,” “flavonoid biosynthesis,” and “phenylpropanoid biosynthesis” are the overrepresented pathways in cassava leaves affected by TSSM, and the enrichment pattern was more significant with an increase in infestation duration (Figure 1C; Supplementary Figures S3A,B). The “phenylpropanoid biosynthesis” and “flavonoid biosynthesis” pathways are closely related to the biosynthesis of proanthocyanidins, tannins, flavones, and flavonols; therefore, we continued to excavate gene-related metabolites at the metabolomic level.

### Two-spotted spider mite infestation elevates the production of specialized defensive metabolites in infested plants

To gain an overview of the metabolome changes induced by TSSM, non-targeted metabolomics analyses of cassava samples was performed using UPLC-QTOF MS (see MetaboLights database, accession ID: MTBLS5093). Overall, a total of 605 metabolites were identified. PCA analysis of the resulting metabolite data revealed a clear separation of the HR and HS groups. However, within the HR and HS groups, the metabolites in samples before mite infestation (0 day) and those in 1 day post-infested samples showed some overlap, wherein they were completely separated in 8 days post-infested samples (Supplementary Figure S1B), implying the hysteresis of metabolite changes compared with the gene transcription. The identified metabolites in different comparisons were ranged from 37 (HR1 vs. HR0) to 225 (HR0 vs. HS0). In addition, the number of DEMs in the HS groups was more abundant than those in the HR groups; this phenomenon was similar to that observed in the transcriptomic analysis (Figure 1D). Heatmap cluster analysis depicted a rather noticeable different pattern in the between-group comparisons (“HR8 vs. HS8,” “HR0 vs. HS0,” and “HR1 vs. HS1”; Figure 1E; Supplementary Figures S2C,D). In consistence with the KEGG enrichment in the transcriptome analysis, the KEGG results of the metabolomes also indicated that “biosynthesis of secondary metabolite,” “flavonoid biosynthesis” and “phenylpropanoid biosynthesis” were usually ranked in the most enriched pathways (Figure 1F; Supplementary Figures S2C,D). However, this enrichment pattern identified for the DEMs was more significant than that identified for the DEGs. In addition, *K*-means analysis showed that the DEMs in flavonoids and phenylpropanoids were mainly enriched in three classes, and those chemicals exhibited a general upward trend between HS and HR samples (Figures 1G–I), while other chemical families in other classes did not show such a coinciding trend, and other classes (class 4–12) did not contain as abundant flavonoid and phenylpropanoid chemicals as class 1–3 (Supplementary Figure S4). These results suggest that there should be a tacit linkage between genes and metabolites in cassava plants during TSSM infestation.

### Integrated transcriptomic and metabolomics analysis reveals coordinated variation of genes and metabolites in specific secondary metabolite pathway

**Figure 2:**
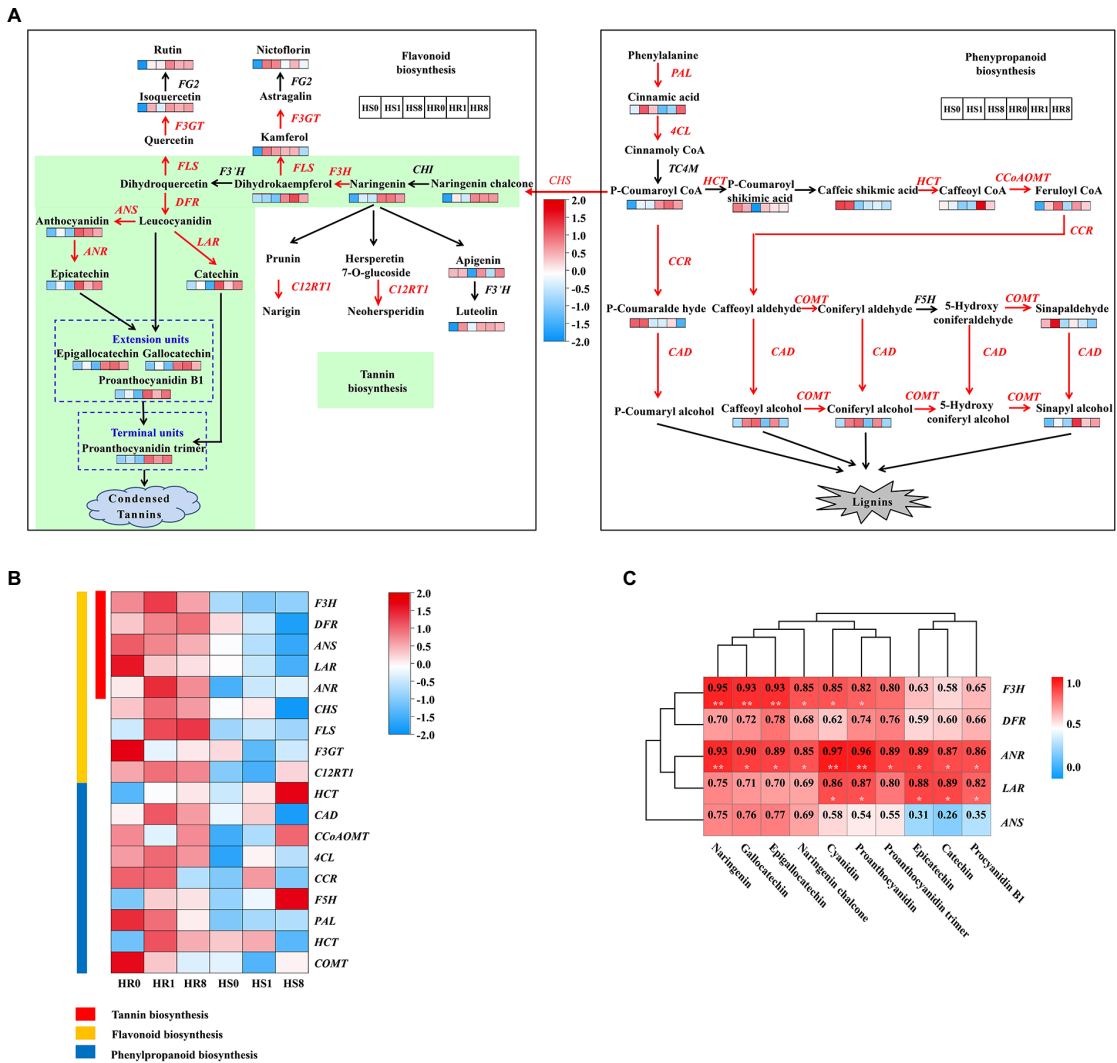
Changes in expression of genes and abundances of metabolites related to flavonoid/phenylpropanoid biosynthesis during TSSM infestation on HR and HS cultivars at different infestation time points [0 d (HR0 and HS0), 1 d (HR1 and HS1), and 8 d (HR8 and HS8)]. **(A)** Summary of pathways of flavonoid/phenylpropanoid biosynthesis. Heatmaps are shown where the abundance of the metabolite changed significantly between the infestation times of HR and HS samples. Genes that were identified as being differentially expressed are indicated in red, definitions of the groups are presented in the black and white frames. **(B)** Heatmap of flavonoid/phenylpropanoid biosynthesis genes significantly affected during TSSM infestation. **(C)** Correlation between the DEGs and DEMs in the tannin biosynthesis pathway. Single asterisk and double asterisk indicated significant (*p* < 0.05) and extremely significant level (*p* < 0.01), respectively.

Our results showed that TSSM infestation triggered direct defense responses in cassava plants, including induced gene expression and metabolite production in the phenylpropanoid and flavonoid pathways. More specifically, mite feeding elevated the expression of genes encoding the key metabolic enzymes such as phenylalanine ammonia-lyase (PAL), 4-coumarate-CoA ligase (4CL), chalcone synthase (CHS), flavanone 3-hydroxylase (F3H), catechol-O-methyl transferase (COMT), dihydroflavonol 4-reductase (DFR), anthocyanidin reductase (ANR) and leucoanthocyanidin reductase (LAR) involved in phenylpropanoid/flavonoid biosynthesis (Figure 2A). Similar to the increasing trend of expression of the genes, the abundance of some characteristic phenylpropanoid compounds such as cinnamic acid, p-coumaroyl-CoA, and sinapyl alcohol, and flavonoid compounds such as catechins, proanthocyanidin dimers, rutin, flavone glycosides, and condensed tannins was drastically increased in TSSM-infested cassava leaves (Figure 2A). In addition, the tannin biosynthesis pathway embedded in the flavonoid pathway showed even more vigorous upregulation in HR samples compared with the HS samples, as almost all the genes and related intermediate metabolites in this pathway were consistently induced (Figures 2A,B). Furthermore, correlation analysis indicated that the transcription of tannin biosynthesis genes (i.e., *F3H*, *DFR*, *ANR*, *ANS*, and *LAR*) and the abundance of most related metabolites (i.e., naringenin, gallocatechin, epigallocatechin, cyanidin, proanthocyanidin, catechin, epicatechin and procyandin B1) showed a positive correlation, particularly for *LAR* and *ANR*, which possessed the highest frequency of highly significant positive correlation (Figure 2C). In tannins biosynthesis pathway, ANR catalyze anthocyanins to form epicatechins, and LAR convert the leucoanthocyanidins into the corresponding catechins *in vitro* (Dixon et al., 2005; Wang et al., 2018a), and then undergo the polymerization step to form condensed tannin (Dixon et al., 2005).

### RT-qPCR and enzymatic analysis showed a coordinate elevation of transcription and activity of tannin biosynthesis genes after TSSM infestation

**Figure 3:**
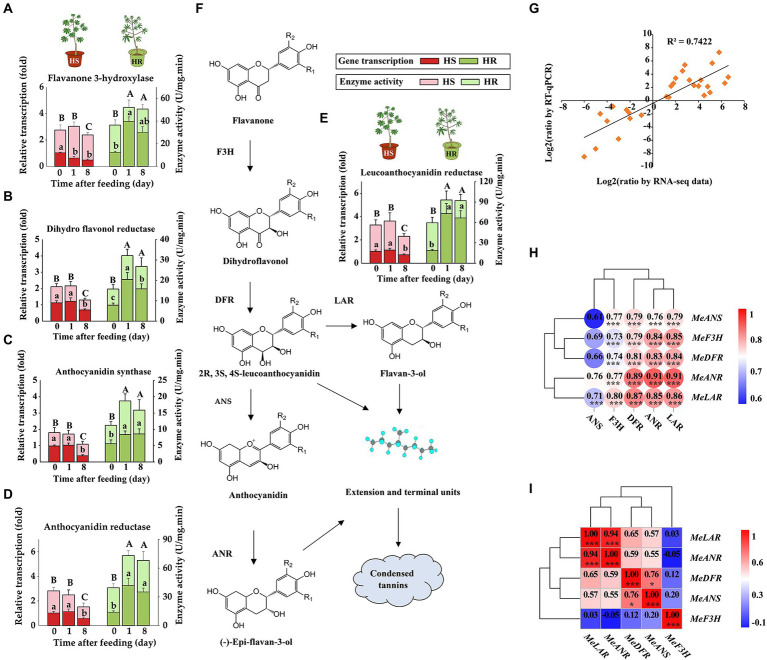
RT-qPCR and enzymatic validation of tannin biosynthesis genes. **(A–F)** Transcription and activity changes of tannin biosynthesis genes in HS and HR cassava plants, different lowercase letters above standard error bars indicate significant differences in gene transcription of different time points within a cultivar, and capital letters indicate significant differences in enzyme activity across cultivars, all analysis was based on one-way analysis of variance (ANOVA) with Tukey’s honestly significant difference (HSD) multiple comparison test (*p* < 0.05): **(A)** F3H, **(B)** DFR, **(C)** ANS, **(D)** ANR, **(E)** LAR. **(F)** The diagram of condensed tannin biosynthesis. **(G)** Correlation between RT-qPCR and RNA-Seq results. **(H)** Correlation between the transcription and activity of enzymes involved in tannin biosynthesis. **(I)** Correlation among the transcription of tannin biosynthesis genes.

In the tannin biosynthesis pathway, the expression of genes encoding key metabolic enzymes such as F3H, DFR, ANS, LAR, and ANR was analyzed using RT-qPCR and enzymatic analysis. The results demonstrated that before TSSM infestation, the enzyme activity between HS and HR plants did not show significant difference, indicating identical constitutive defense ability. However, after TSSM infestation, the transcription and enzyme activities significantly increase in HR plants, while the HS plants suffered both transcription and enzyme activity suppression with mite infestation over time (Figures 3A–F), which probably indicated that the TSSM infestation may trigger the tannins biosynthesis pathway in HR plant but suppress this pathway in HS plant. In addition, the RT-qPCR results showed a high correlation with the transcriptomic analysis (*R*^2^ = 0.7422; Figure 3G), moreover, the transcription of tannin biosynthesis genes showed a positive correlation with the activity of their coding enzymes (Figure 3H). Especially, the transcription of *MeLAR* and *MeANR* showed high positive correlation with each other as well as the other major tannin biosynthesis genes (i.e., *MeDFR, MeANS*; Figure 3I). We speculated that these two genes may play key role in tannins synthesis and attribute to cassava resistance to TSSM. Therefore, *MeLAR* and *MeANR* were used for transformation in mite-susceptible cassava to further validate their potential resistant function.

### Cassava plants overexpressing *MeLAR* or *MeANR* enhance resistance to TSSM

**Figure 4:**
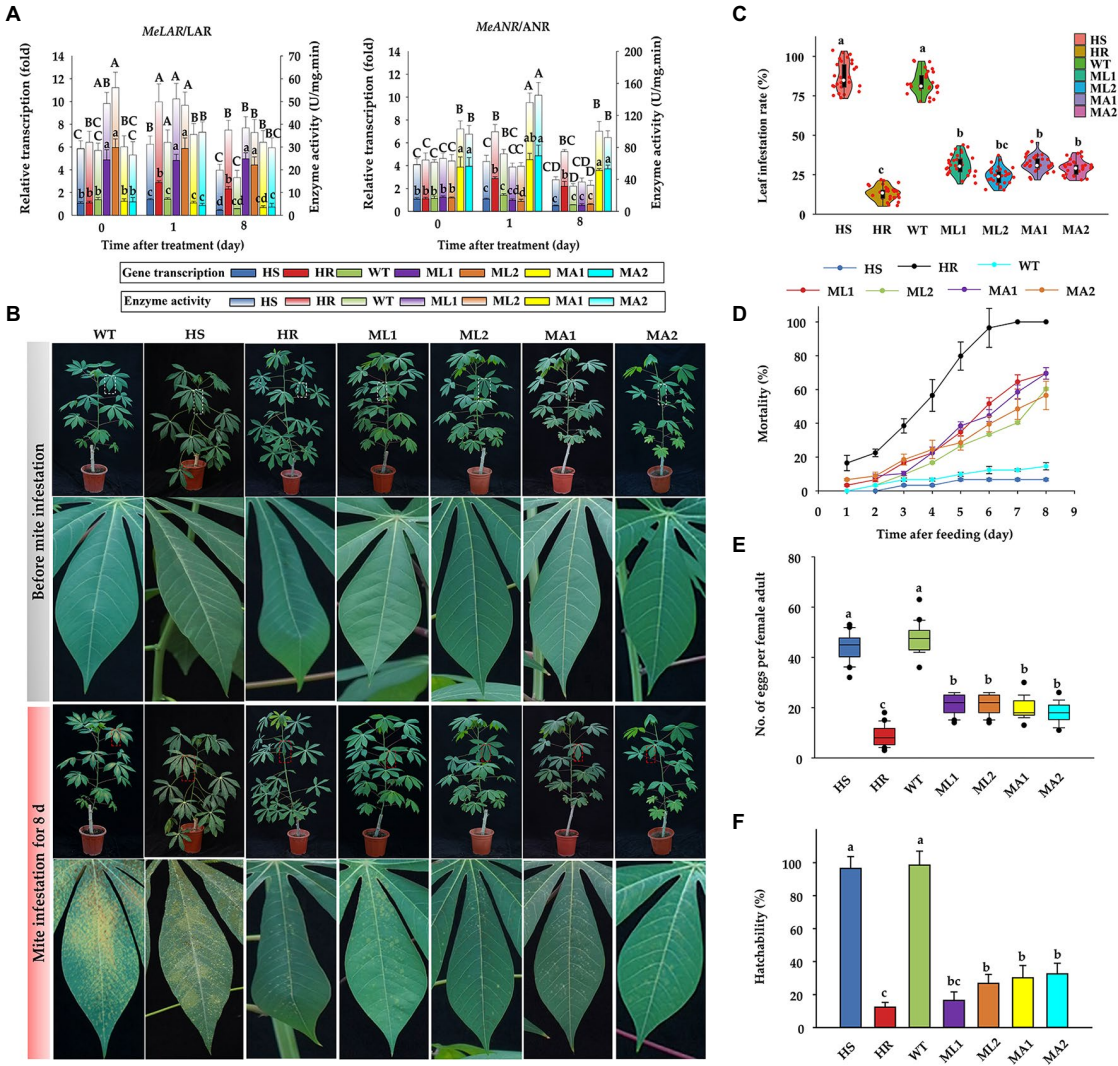
Performance of transgenic cassava lines against TSSM infestation. **(A)** Alterations in the relative transcription and enzyme activities of *LAR* and *ANR* at different time points of TSSM infestation in HR, HS, WT, and transgenic cassava plants (ML1 and ML2 stand for transgenic lines overexpressing *LAR* gene, and MA1 and MA2 stand for transgenic lines overexpressing *ANR* gene, respectively), different capital and lowercase letters above standard error bars indicate significant differences in transcription and activity of *LAR* and *ANR* across different cassava plants based on one-way analysis of variance (ANOVA) with Tukey’s honestly significant difference (HSD) multiple comparison test (*p* < 0.05), respectively. **(B)** The TSSM infestation symptom of HR, HS, WT, and transgenic cassava plants. The “zoom in” areas of plants before mite infestation and mite infestation for 8 days were indicated by white dashed boxes and red dashed boxes, respectively. **(C)** The leaf infestation rate of cassava plants after TSSM feeding, different letters indicate significant differences based on one-way analysis of variance (ANOVA) with Tukey’s honestly significant difference (HSD) multiple comparison test (*p* < 0.05). **(D)** Effect on mortality of TSSM after feeding HR, HS, WT, and transgenic cassava plants. **(E)** Effect on the fecundity of TSSM after feeding on HR, HS, WT, and transgenic plants. **(F)** Effect on the hatchability of TSSM after feeding on HR, HS, WT, and transgenic plants.

The CDS of *MeLAR* and *MeANR* were successfully amplified (Supplementary Figures S5A,B) and the corresponding transgenic vectors with the insertion of the individual gene (either *MeLAR* or *MeANR*) fragment were constructed (Supplementary Figures S5C,D). Following the transformation procedure (Xu et al., 2013), we successfully established the cassava lines overexpressing *MeLAR* (ML1 and ML2 lines) and *MeANR* (MA1 and MA2 lines; Supplementary Figure S6A), as validated by Southern blot (Supplementary Figures S6B,C) and PCR detection (Supplementary Figure S6D). The transgenic cassava lines exhibited significantly higher transcription of *LAR* and *ANR* than WT and HS plants (approximately 4–6 fold), moreover, with the development of mite infestation, the transcription of *LAR* and *ANR* in transgenic lines were basically significantly higher than the WT and HS plants. In addition, compared with the enzyme activity, the gene transcription of *LAR* and *ANR* were more stable under mite infestation (Figure 4A), as the enzyme activity of LAR and ANR in transgenic lines would decrease a bit on 8-day-post infestation, while the transcriptions still stay unchanged, in comparison, both the transcription and enzyme activity in WT and HS plants suffered a significant suppression. Evaluation of these transgenic lines against TSSM infestation showed that the transgenic lines did not show serious infestation symptoms both in laboratory (Figure 4B) and field conditions (Supplementary Figure S7A); the damaged leaf area ranged from 17.3 to 29.2% in the greenhouse, while it was over 85% in WT plants (Figure 4C), indicating the significant improvement of TSSM-resistant level for the transgenic lines. The resistance performance under field conditions were concordant with the laboratory performance, with the mite infestation index ranging from 12.8 to 23.5% (Supplementary Figure S7B), which suggested that these transgenic cassava plants were “resistant” to TSSM according to our established evaluation method (Lu et al., 2017), similarly, the WT plant was also severely infested by TSSM in the field (Supplementary Figure S7B). Although the mortality rate of TSSM feeding on transgenic cassava lines did not reach the level as obtained for HR plants, it was higher than that of TSSM feeding on WT as well as HS plants (Figure 4D). In addition, the fecundity (Figure 4E) and hatchability (Figure 4F) of TSSM reared on the transgenic lines was significantly suppressed than that of TSSM reared on WT and HS plants. These results indicate that cassava plants overexpressing *MeLAR* or *MeANR* are resistant to TSSM.

### Different forms of tannins possess different bioactivity to TSSM

**Figure 5:**
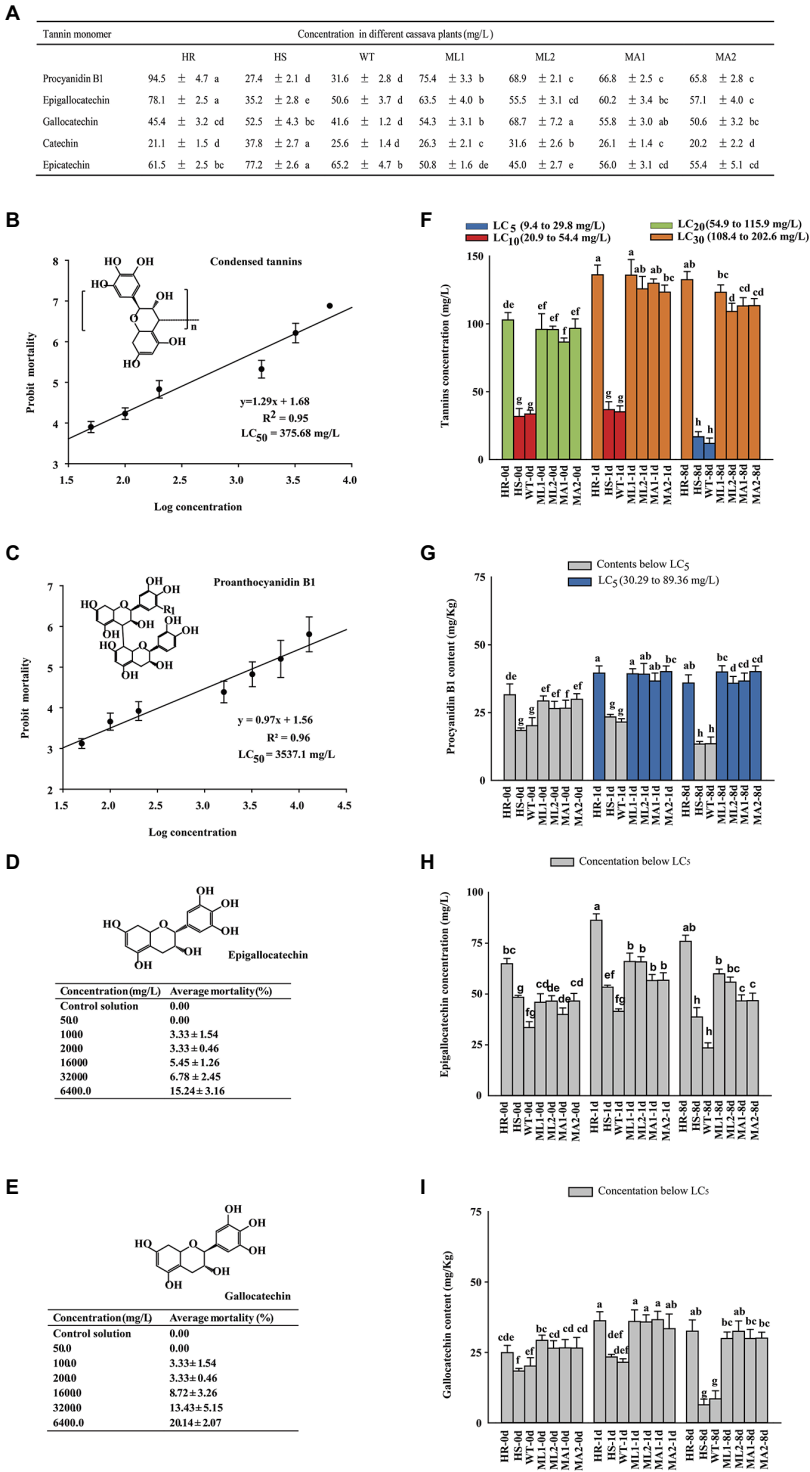
The bioactivity of different forms of tannins on TSSM and the concentration changes in cassava plants under TSSM infestation. **(A)** The concentration of terminal and extension units of condensed tannins of cassava leaves was determined using HPLC following thiolysis degradation. **(B,C)** The probit mortality analysis of **(B)** total condensed tannins and **(C)** proanthocyanidin B1 on TSSM. **(D,E)** The mortality of TSSM treated with different concentrations of **(D)** epigallocatechin and **(E)** gallocatechin. **(F–I)** The concentration changes of different forms of tannins in different cassava plants under TSSM infestation: **(F)** The total condensed tannins, **(G)** Proanthocyanidin B1, **(H)** Epigallocatechin, and **(I)** Gallocatechin. Legends and columns with the same color indicate the concentrations of tested compounds in cassava plants are equal to their toxicity indexes (i.e., LC_5_, LC_10_, LC_20_ or LC_30_). In addition, different letters indicate significant differences based on one-way analysis of variance (ANOVA) with Tukey’s honestly significant difference (HSD) multiple comparison test (*p* < 0.05).

Reversed-phase HPLC analysis identified catechin, epicatechin, gallocatechin, epigallocatechin, and proanthocyanidin B1 as the condensed tannin degradation products (Figure 5A; Supplementary Figure S8), indicating that they were the extension and terminal units in the condensed tannins of cassava leaves. The condensed tannin concentration and its extension and terminal unit compounds were higher in the HR plants than in the HS plants, indicating the difference in the constitutive defense against TSSM infestation (Figure 5A). The total extracted condensed tannin, together with its different forms of precursors (the terminal and extension units, i.e., procyanidin B1, epigallocatechin, and gallocatechin), were used to check their lethal effect on TSSM using soaking method (Supplementary Figure S9). The concentrations of these compounds in different cassava plants were measured after mite infestation. The bioassay results showed that total condensed tannin displayed the highest bioactivity, with an LC_50_ level of 375.68 mg/l (Figure 5B), followed by procyanidin B1, with an LC_50_ level of 3537.10 mg/l (Figure 5C). Furthermore, the epigallocatechin and gallocatechin showed pretty low bioactivity—sublethal effects can only be seen at very high concentrations (6,400 mg/l, mortalities were all below 20%; Figures 5D,E). In addition, the concentrations of these compounds at different time points of TSSM infestation were also tested. Figures 5F,I shows that the four compounds (i.e., total condensed tannin, procyanidin B1, epigallocatechin, and gallocatechin) displayed a consistent change pattern. On the one hand, before infestation, the contents of these compounds were higher in the HR plant and four transgenic lines compared with those in the WT and HS plants. On the other hand, the contents of the four compounds increased during the initial days of mite infestation but reduced significantly on 8 day-post-infestation in WT and HS plants. Furthermore, comparison of the lethal concentrations acquired from the bioassay, only the contents of total tannin in all cassava plants, and the contents of procyanidin B1 in 1 day-infested HR and transgenic cassava lines were detected beyond LC_5_ (Figures 5F,G), while the contents of epigallocatechin and gallocatechin were far below their corresponding lethal concentrations (Figures 5H,I). Nevertheless, all the tested tannin-related compounds exhibited a sub-lethal effect on TSSM (the highest total condensed tannin concentration equal to LC_30_; Figure 5F).

## Discussion

Several studies had illustrated that metabolites such as flavonoids (Gazola et al., 2019; Yang et al., 2020), phenylpropanoid (Yang et al., 2019) and total tannin (Osipitan et al., 2015) were responsible for cassava resistance to insect or mite species. However, there was a lack of further step to excavate and validate the specific compounds that definitely possessed bioactivity. We assumed that among a large number of metabolites, only a few chemicals accounted for plant resistance, and by employing a highly resistant and highly susceptible cassava cultivars, the opportunity of targeting putative plant resistance-related genes and chemicals would largely increase. In this study, the transcriptome and metabolome generated a huge of the DEGs and DEMs, and the enrichment analysis indicated abundant DEGs and DEMs were concentrated in phenylpropanoid/flavonoid pathways, in particularly, the tannin synthesis pathway, which embedded in the flavonoid pathways, exhibiting that all the genes and metabolites were coordinately upregulated, as depicted by the transcriptomic and metabolomic analyses. Further RT-qPCR validated that the transcription of these genes was significantly increased after TSSM infestation. The analyses identified *MeLAR* and *MeANR* as the two key genes showing the most significant expression. Transgenic cassava lines overexpressing these two genes were used to assess their resistance to TSSM. The results showed that overproduction of tannins could be seen in the transgenic cassava lines. In addition, the transgenic cassava lines showed significantly enhanced resistance to TSSM, as suggested by the leaf damage, mortality, fecundity, and hatchability of TSSM (Figure 4).

### Genes and metabolites involved in phenylpropanoid/flavonoid biosynthesis are upregulated after TSSM infestation

The present study, employing integrated transcriptomic and metabolomics approaches, unraveled a gene-metabolite network and provided insights into understanding the mechanism underlying TSSM-resistance in cassava. This study demonstrated that most genes and related metabolites involved in phenylpropanoid/flavonoid biosynthesis were co-upregulated, indicating the critical role of these two pathways in TSSM defense. More specifically, mite feeding elevated the expression of genes encoding key metabolic enzymes (PAL, 4CL, CHS, F3H, COMT, DFR, ANR, and LAR) involved in phenylpropanoid/flavonoid biosynthesis (Figure 2A). Furthermore, the increased abundance of phenylpropanoid and flavonoid compounds suggested the pivotal role of these compounds in defense against TSSM. Concordant with the present study, several other studies reporting the plant-insect interactions have demonstrated the importance of these two pathways in defending plants against piercing-sucking insect herbivores (Zhao et al., 2020) and chewing insects (Wang et al., 2016). These two pathways have also been demonstrated to be involved in TSSM defense in several crop plants, including tomato (Weinblum et al., 2021), pepper (Zhang et al., 2019), bean (Scott et al., 2021), citrus (Agut et al., 2014) and cassava (Yang et al., 2020). Taken together, it appears that the general upregulation of the phenylpropanoid and flavonoid pathways is a common strategy employed by different plants encountering insect attacks, regardless of the feeding mode. However, as it is well known that hundreds of phenylpropanoid and flavonoid compounds are significantly expressed in plants under insect pest stress, further determination of the specific compounds that play a leading role in plant resistance is still tricky.

### The tannin biosynthesis pathway embedded in the flavonoid pathway showed even more vigorous and prevailing induction of both genes and metabolites in HR cassava cultivars compared with HS cultivars

Several studies have aimed to illustrate the functions of certain flavonoid compounds. For instance, rutin, when applied at concentrations between 10^−4^ and 10^−5^ M, stimulated the feeding of several species of *Spodoptera* and *Helicoverpa* but deterred the feeding of the same insects at higher concentrations (Schweiger et al., 2014). Likewise, at higher concentrations, the flavonoids isorhamnetin 3-sophoroside-7-glucoside and kaempferol 3, 7-diglucoside acted as effective feeding deterrents against *Mamestra configurata* (Walk.; Züst and Agrawal, 2017). A recent study in cassava using proteomic and transcript analyses has demonstrated that the resistant cassava cultivar XX048 to *T. cinnabarinus* was significantly enriched in the biosynthesis of flavonoids (phenolic compounds) compared to the susceptible cultivar GR4 (Yang et al., 2020); however, the key functional compounds in cassava remain unclear. Here, we found that the tannin biosynthesis pathway embedded in the flavonoid pathway showed even more vigorous and prevailing induction of both genes and metabolites in HR cassava cultivars compared with HS cultivars. Among several tannin synthesis genes, the *LAR* and *ANR* genes showed a specific parallel tendency in three ways. First, compared to other genes in the tannin pathway, the transcriptional upregulation of these two genes was positively correlated with the accumulation of several intermediate metabolites (Figure 2C). Second, the transcription of *LAR* and *ANR* genes changed in coordination with their corresponding coding enzymes, indicating the consistency in their transcriptional and translational processing (Figure 3H). Third, the transcription of *LAR* and *ANR* genes showed the highest correlation among the five tannin synthesis genes, as depicted by correlation analysis (Figure 3I), we assumed that these two genes work separately in the last step of tannin synthesis (Figure 3F) and was highly correlated.

### Overexpression of *LAR* and *ANR* confers resistance to TSSM in cassava by increasing the content of specific tannin compounds

Considering the potential critical function of these two genes in tannin synthesis and mite-resistance formatting, transgenic cassava plants overexpressing *LAR* or *ANR* were used to evaluate resistance to TSSM. We found that all the transgenic cassava lines acquired both constitutive and inducible resistance to TSSM, and these cassava lines could significantly inhibit the development and reproduction of TSSM and exhibited comparable phenotypic resistance to the HR plants. Manipulation of *LAR* and *ANR* expression has also been conducted in other plant species. For example, overexpression of the *Malus crabapple* genes *MrLAR1, 2* and *MrANR1, 2* in tobacco (*Nicotiana tabacum*) promoted the accumulation of tannins, whereas transient silencing of their expression in crabapple resulted in reduced PA levels. In addition, silencing banana (*Musa acuminata*) *MaANR1* reduced tannin content in transgenic banana plants (Ghag et al., 2015). Transgenic poplar (*Populus trichocarpa*) plants overexpressing *PtrLAR3* displayed a significant elevation in tannin levels and reduced their disease symptoms compared to WT plants (Li et al., 2012). The transgenic tobacco overexpressing tea (*Camellia sinensis*) *CsANR* gene also showed resistance against infestation by a tobacco leaf cutworm, *Spodoptera litura*. In addition, some studies have demonstrated that modulation of specific transcription factors also leads to a dramatic accumulation of tannins, but not necessarily other flavonoids (Wang et al., 2018b). Although several tannin-overproducing transgenic plants have been developed, their performance in insect resistance has seldom been investigated. This study is the first attempt to confirm that overexpressing *MeLAR* or *MeANR* confers cassava resistance to TSSM. However, how these genes are regulated remains unclear in cassava. There are several studies demonstrated that transcription factor such as MYB can regulate the expression of tannin biosynthesis genes including ANR and LAR in different plants, i.e., citrus (He et al., 2022), alfalfa (Liu et al., 2014) and grape (Cheng et al., 2021), thus, we assumed that this transcription factor might possess similar function in cassava, but future study is warranted to support this viewpoint.

Condensed tannins can defend leaves against insect herbivores through deterrence and/or toxicity (Barbehenn and Peter Constabel, 2011). Insect damage and wounding can have strong stimulatory effects on tannin production in some plants, suggesting that tannin synthesis contributes to the induced defense. The induction of tannins by herbivory has been reported in several tree species, including *Pinus sylvestris* (Roitto et al., 2008), *Populus species* (Arnold and Schultz, 2002), and Quercus species (Rossi et al., 2004). As condensed tannins are chemically diverse and multifunctional compounds, different forms of tannins may have different biochemical effects on insect herbivores (Barbehenn and Peter Constabel, 2011); however, little work has been done to support this assumption. In this study, metabolome analysis first identified some different tannin compounds, which were then validated using HPLC following thiolysis degradation. The bioassay of those compounds further suggested that the total condensed tannins showed the best lethal effect to TSSM, followed by the tannin dimer proanthocyanidin B1, which was approximately ten-times lower effect than the former one, in comparison, the tannin monomer epigallocatechin and gallocatechin showed pretty low bioactivity to TSSM. This phenomenon confirmed our hypothesis that certain compounds in specific metabolites family play dominant role in defense against insect pest. Moreover, compared with the WT plants, the concentrations of these four compounds in transgenic cassava lines were increased and inducible after TSSM feeding, while the concentrations of catechin and epicatechin did not change. Similar observations of no significant effect of tea green leafhopper attack on alteration of catechin and total catechin contents in dissected tea leaves has been reported in a recent study (Liao et al., 2019). The HS plants, like WT, failed to induce tannin compounds, which probably explains why the inducibility of tannin content might differentiate the resistance levels of plants. This hypothesis is supported by several studies in trees, such as *Quercus serratus* (Kouki et al., 2005) and silver birch (*Betula pendula*; Keinänen et al., 1999), in which tannin levels were not altered by damage or herbivory, suggesting that inducibility is not a universal response of tannin-accumulating plants. According to previous studies, tannins are feeding deterrents to many invertebrate herbivores including insects. When tannin is ingested by insect, fatal lesions were produced in their midguts (Bernays and Chamberlain, 1980), and lesions were thought to be due to tannins permeating the peritrophic envelopes, and then binding with membranes of the midgut epithelium (Barbehenn, 2001). In addition, tannin may act as prooxidants in some insects (Barbehenn and Peter Constabel, 2011). For example, tannin-induced gut lesions are potentially caused by oxidative stress in poorly adapted insect species. However, whether this mechanism also fits the situation of TSSM remains mysterious, there is a need to conduct delicate study in the future.

Nevertheless, different tannin compounds only cause sub-lethal effects on TSSM, and some questions remain to be answered regarding whether other compounds from other pathway also showed comparable or even better anti-TSSM performance, for instance, in present study we found lignin, the final product of phenylpropanoid metabolism, also exhibited a coordinated upregulation of genes and metabolites in the pathway like tannin (Figure 2A), and moreover, how the enhanced production of tannin chemicals or other compounds modulates resistance to TSSM and whether they act additively or synergistically in feeding deterrence. In addition, more efforts should be made to elucidate the regulatory network of tannins synthesis in cassava and decipher how these tannin compounds inhibit the development and reproduction of TSSM.

## Conclusion

**Figure 6:**
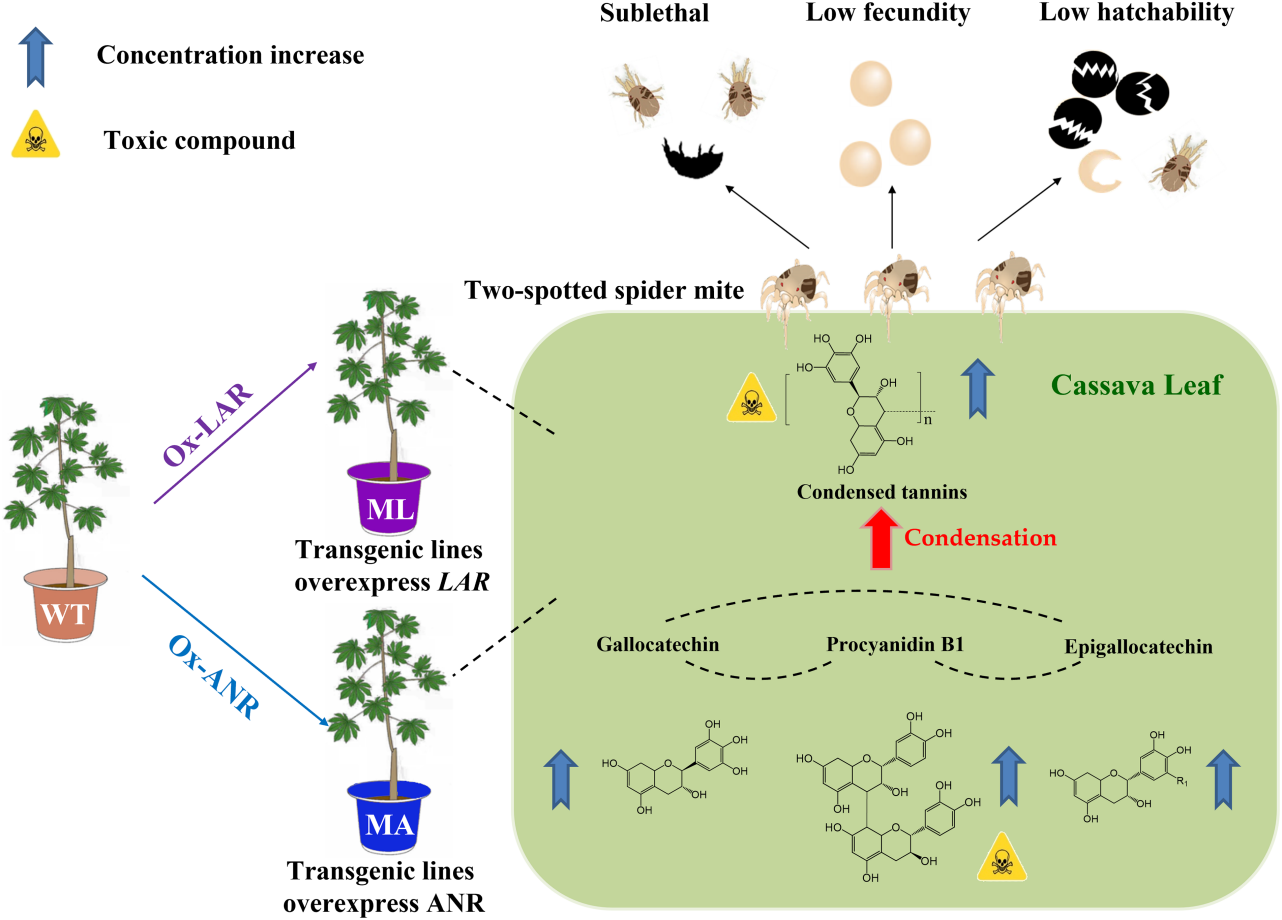
Summary of the dissection of candidate TSSM resistant genes *MeLAR* and *MeANR* and validation of their role in overproducing tannins and confers cassava resistance to TSSM.

Here we elucidates that overexpression of LAR or ANR in cassava plants can elevates the content of the toxic chemical—condensed tannin, which confers cassava resistance to TSSM. The present work validates our hypothesis that a few rather than numerous genes and metabolites can shape cassava resistance to TSSM, the possible mechanism mentioned above was depicted in Figure 6. This study demonstrates the importance of tannins and their key biosynthesis genes in plants for defending piercing-sucking herbivores, and provides insights into potential genes for the molecular breeding of pest-resistant cassava plants.

## Data availability statement

The datasets presented in this study can be found in online repositories. The names of the repository/repositories and accession number(s) can be found at: https://www.ncbi.nlm.nih.gov/, PRJNA822050.

## Author contributions

XLia, QC, and KL planned and designed research and experiments. XLiu, HZ, KL, HW, ZH, MW, XY, JS, YQ, XZ, and YZ performed laboratory experiments and analyzed data. XLia, QC, CW, and YL wrote and edited the paper. XLia, QC, and SC acquired the funds. All authors contributed to the article and approved the submitted version.

## Funding

This work was supported by National Key R&D Program of China (2019YFD1000500), China Agriculture Research System (CARS-11-HNCQ), The NanFeng earmarked fund of Ministry of Agriculture and Rural Affairs of China (NFZX-2021), and Hainan Major Science and Technology Project (no. ZDKJ202002).

## Conflict of interest

The authors declare that the research was conducted in the absence of any commercial or financial relationships that could be construed as a potential conflict of interest.

## Publisher’s note

All claims expressed in this article are solely those of the authors and do not necessarily represent those of their affiliated organizations, or those of the publisher, the editors and the reviewers. Any product that may be evaluated in this article, or claim that may be made by its manufacturer, is not guaranteed or endorsed by the publisher.

## Footnotes

^1^ http://www.bioinformatics.babraham.ac.uk/projects/fastqc/

^2^ https://phytozome.jgi.doe.gov/pz/portal.html
