# Supplementary material for: Overexpression of leucoanthocyanidin reductase or anthocyanidin reductase elevates tannins content and confers cassava resistance to two-spotted spider mite
Source: Front Plant Sci. 2022 Aug 18;13:994866. doi: 10.3389/fpls.2022.994866 (PMC9433999; doi:10.3389/fpls.2022.994866)
Supplement: Supplementary file 1 [file Data_Sheet_1.PDF]

Supplementary Material

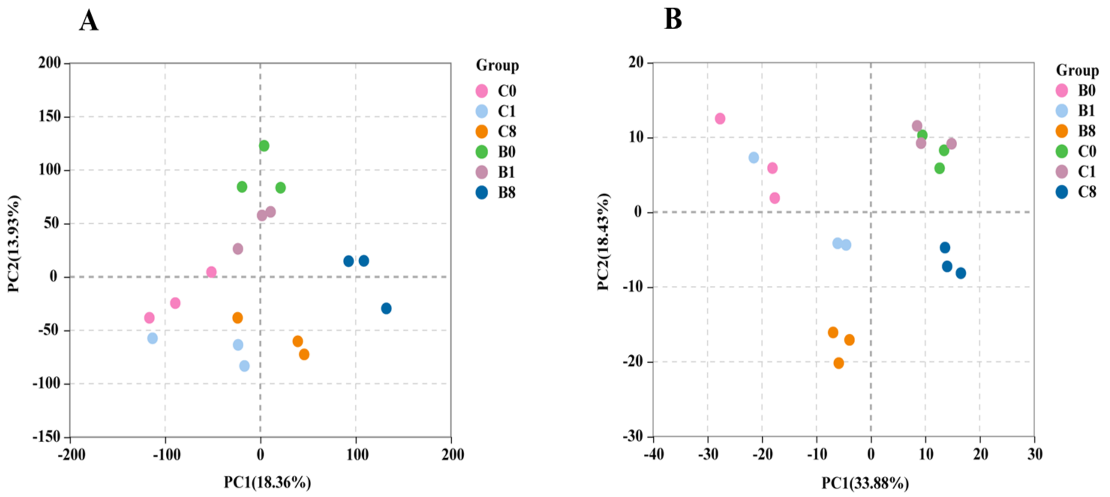

Figure S1 PCA analysis of the (**A**) transcriptome and (**B**) metabolome of C1115 and BRA900 at different time points of infestation (0, 1, and 8 d).

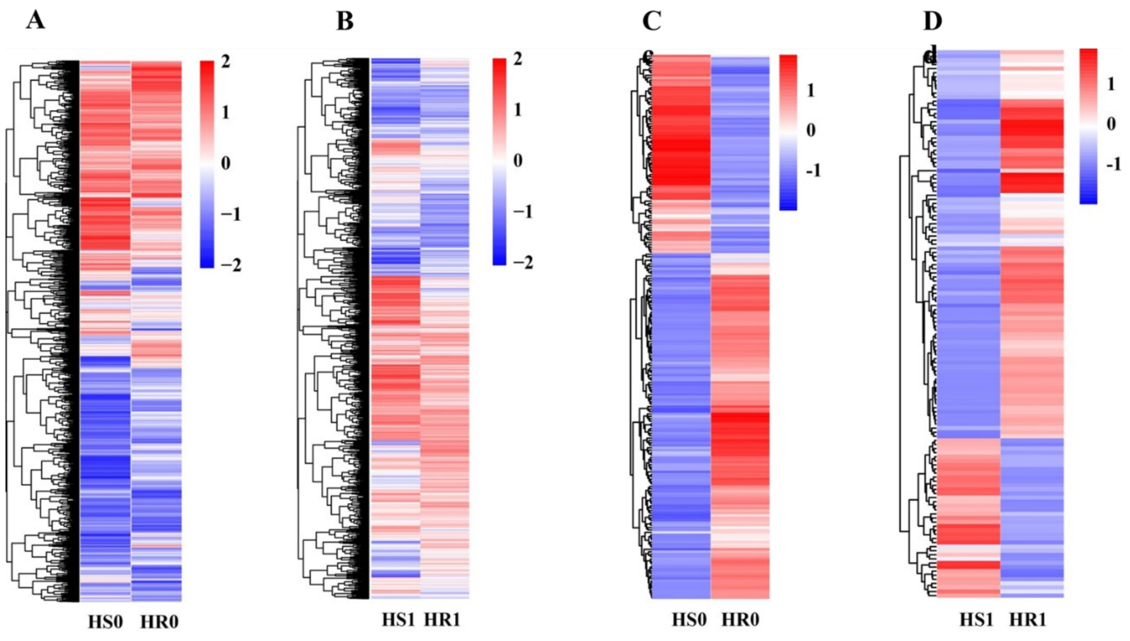

Figure S2 Heatmap cluster analysis of DEGs and DEMs in different HR and HS comparison groups. (**A**–**B**) Heatmap cluster analysis of DEGs (**A**) HS0 and HR0 and (**B**) HS1 and HR1 groups. (**C**–**D**) Heatmap cluster analysis of DEMs between (**C**) HS0 and HR0 and (**D**) HS1 and HR1 groups.

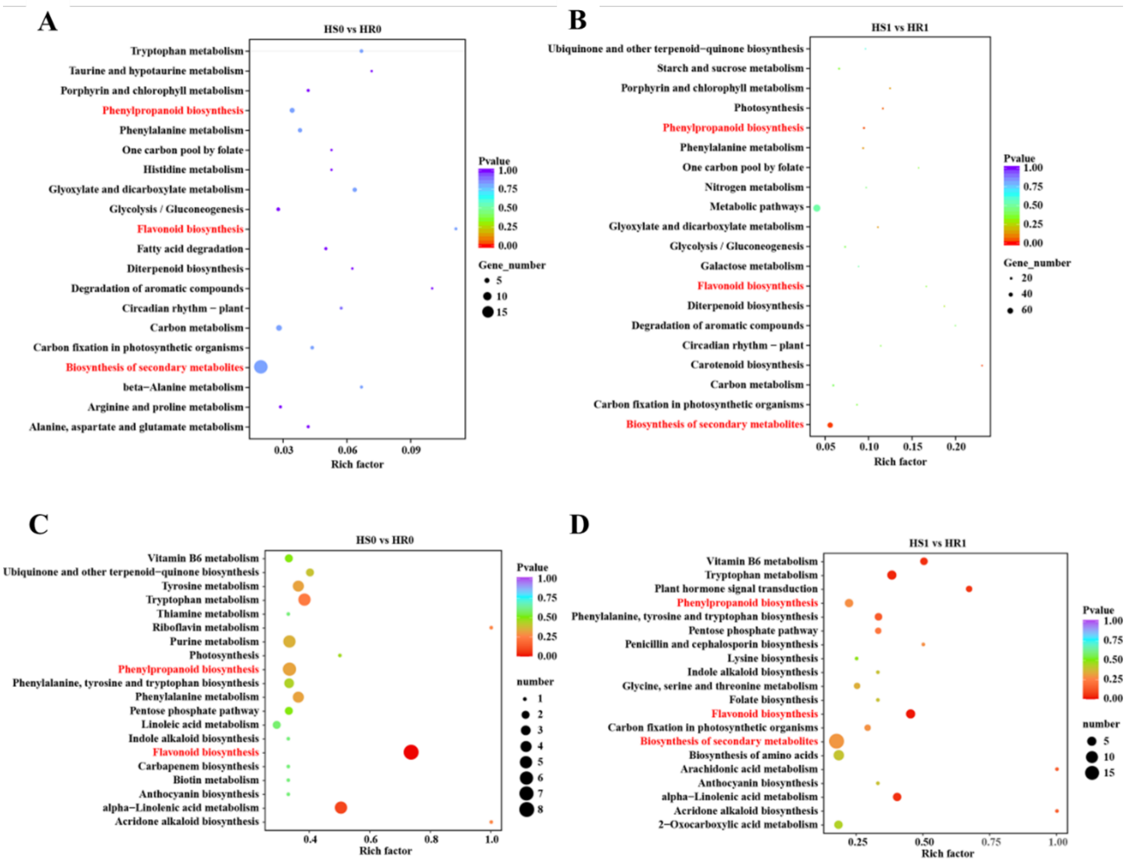

Figure S3 The KEGG enrichment analysis of DEGs and DEMs in different HR and HS comparison groups. (**A**–**B**) KEGG enrichment analysis of DEGs between (**A**) HS0 and HR0 and (**B**) HS1 and HR1 groups. (**C**–**D**) KEGG enrichment analysis of DEMs between (**C**) HS0 and HR0 and (**D**) HS1 and HR1 groups.

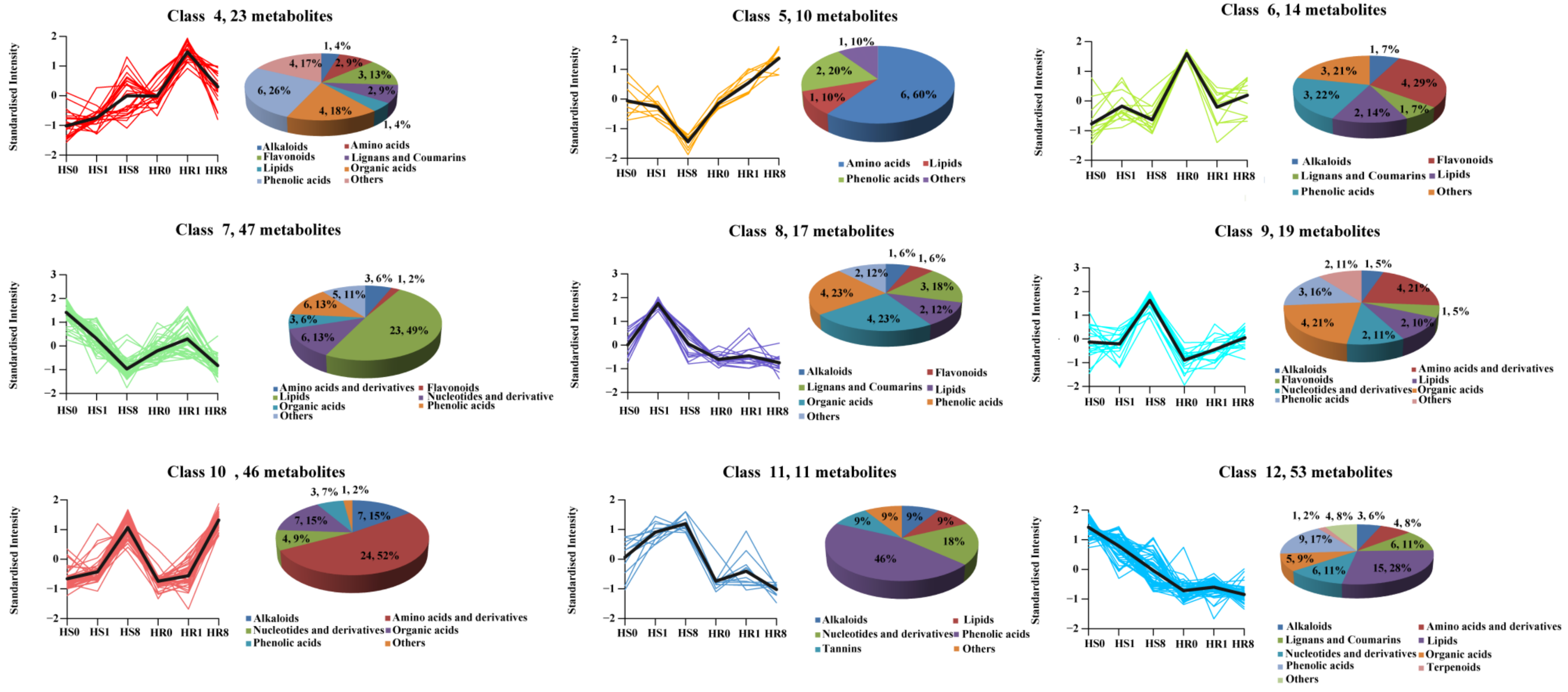

Figure S4 The representative DEMs classes and the proportion of different metabolites families in HS and HR cassava plant samples.

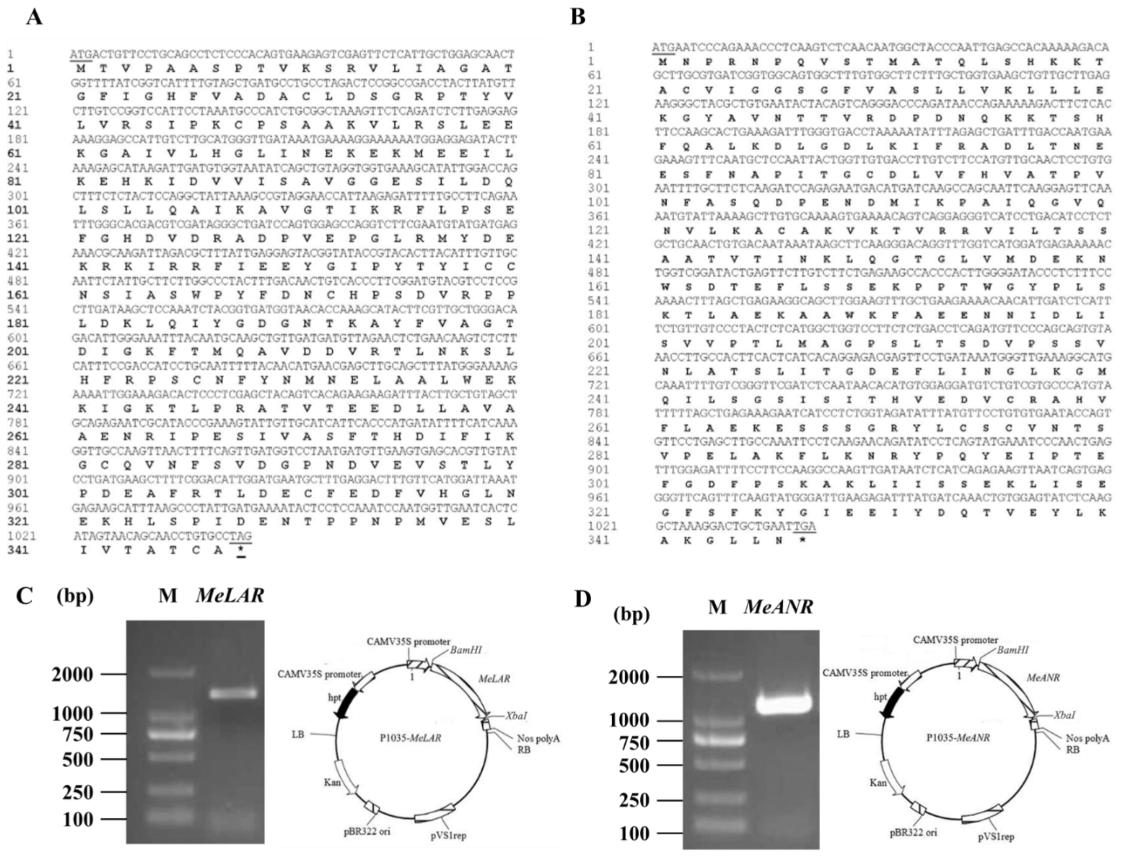

Figure S5 Cloning of cassava *MeLAR* and *MeANR* genes. (**A**–**B**) The coding sequences and deduced amino acid sequences of (**A**) *MeLAR* and (**B**) *MeANR* genes. The amino acid sequence is numbered from the start of its predicted mature protein. The start codon ATG is shown in bold, and the stop codon at the end of the coding region is underlined and marked with an asterisk. (**C**–**D**) The PCR products and the agrobacterium vectors for transformation of *MeLAR* (**C**) and *MeANR* (**D**) genes.

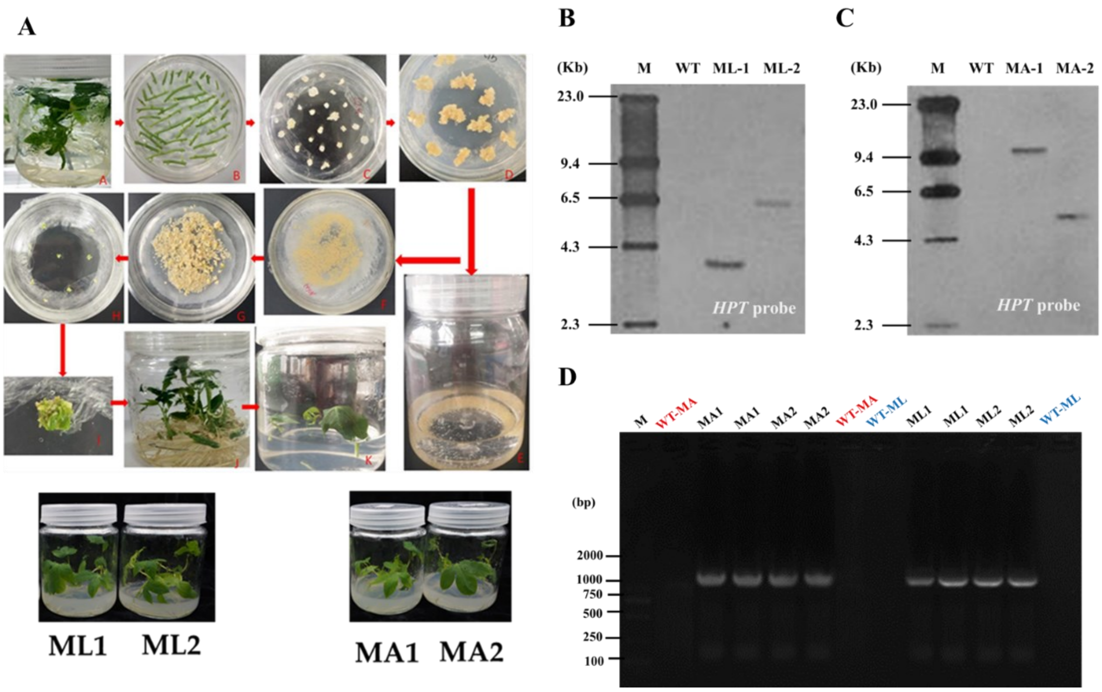

Figure S6 Development and molecular detection of transgenic cassava lines overexpressing *MeLAR* or *MeANR*. (**A**) Development of transgenic cassava overexpressing *MeLAR* or *MeANR*. Southern blot analysis of transgenic line overexpressing (**B**) *MeLAR* and (**C**) *MeANR*. PCR detection of the transformation of (**D**) *MeLAR* and*MeANR*.

A

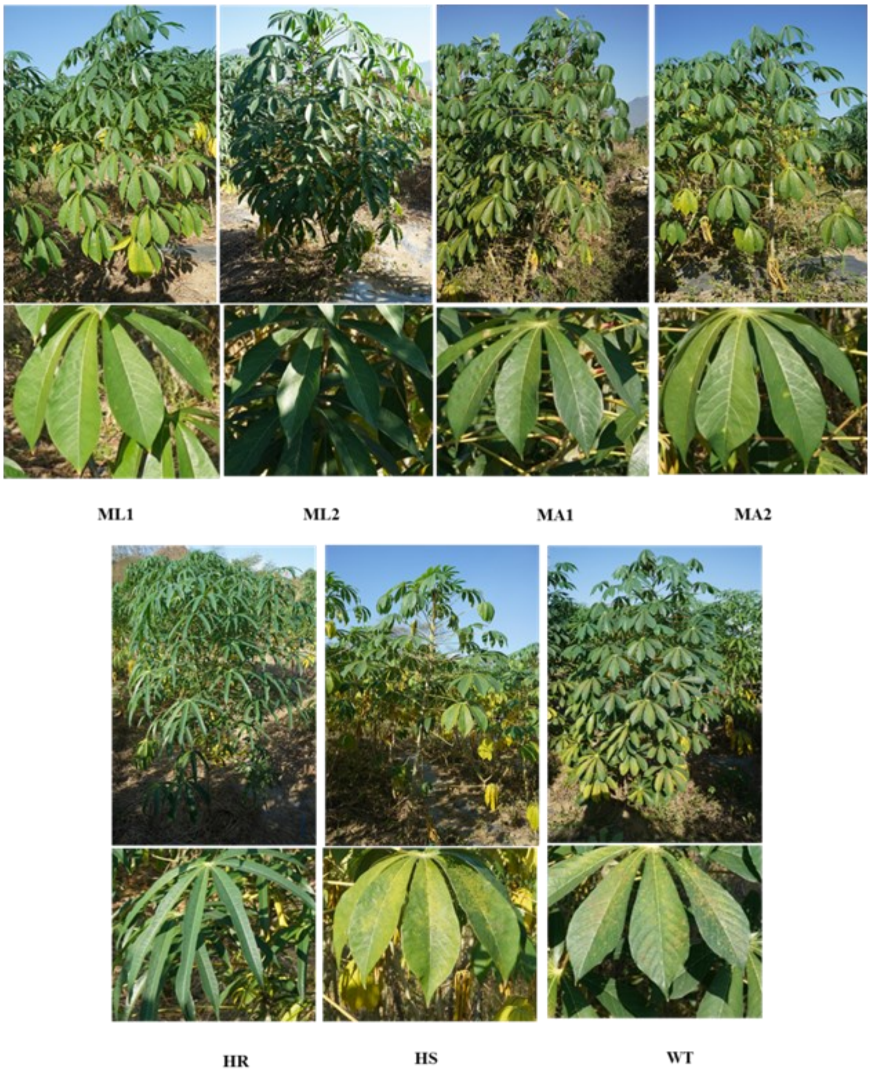

B

Cassava plants	Mite damage index (%)	Resistance level
ML1	12.8	R
ML2	18.2	R
MA1	20.9	R
MA2	23.5	R
WT	85.6	S
HS	94.8	HS
HR	**8.7**	HR

Figure S7 The performance of transgenic cassava lines against TSSM infestation in the field. (**A**) The infestation symptom of transgenic cassava lines in the field. (**B**) Resistance scoring of transgenic cassava lines to TSSM in the field.

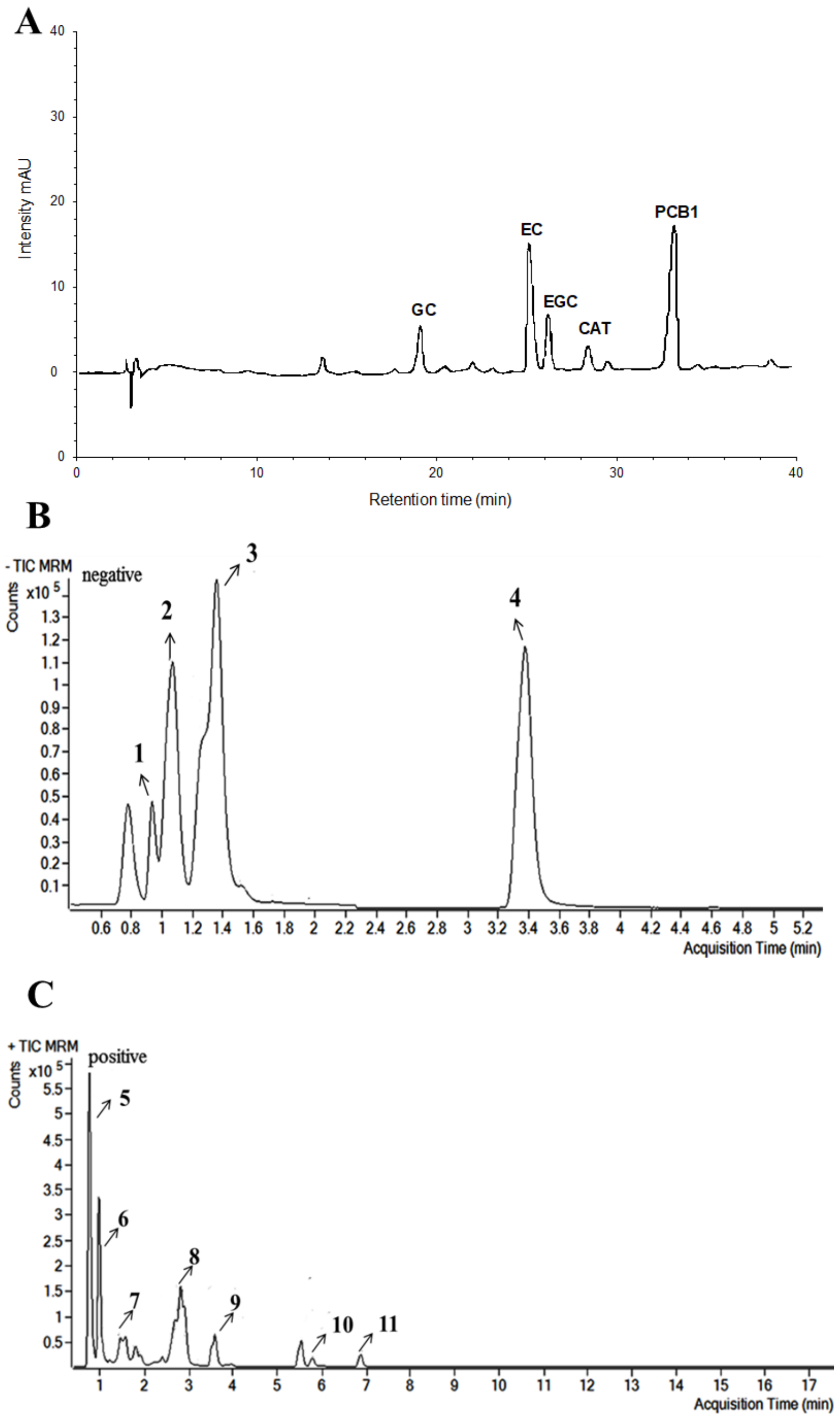

Figure S8 Chromatographic analysis of different forms of tannins in cassava leaves. (**A**) Reversed-phase HPLC chromatograms of condensed tannins from cassava leaves degraded in the presence of cysteamine. EC, epicatechin; CAT, catechin; EGC, epigallocatechin; GC, gallocatechin; PCB1, procyanidin B1. (**B**–**C**) General ion flow chromatographic separation of tannins, (**B**) negative, and (**C**) positive. 1. rutin, 2. kaempferol, 3. dihydrokaempferol, 4. quercetin, 5. dihydroquercetin; 6. catechin, 7. epicatechin gallate, and 8. catechin gallate, 9. epigallocatechin, 10. gallocatechin, 11. procyanidin B1.

A

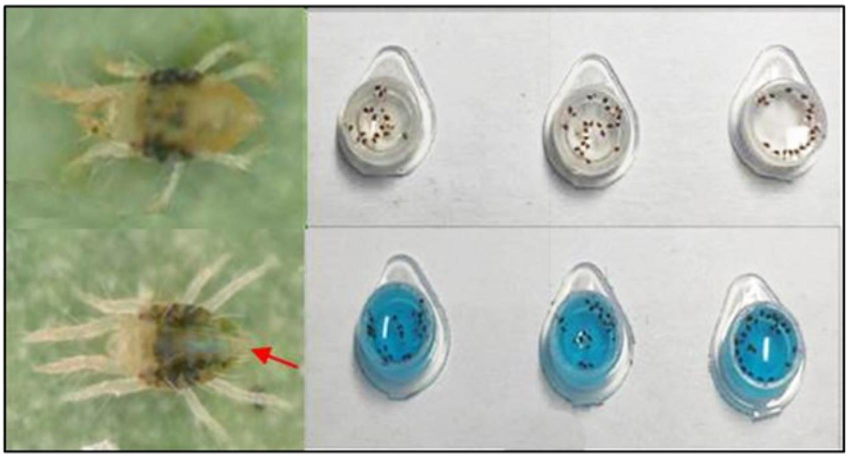

B

Chemical name	CAS No.	Molecular weight	Structure
Condensed tannins	none	> 600	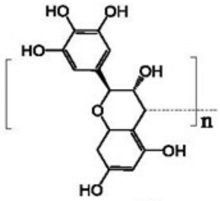
Procyanidin B1	20315-25-7	578.14	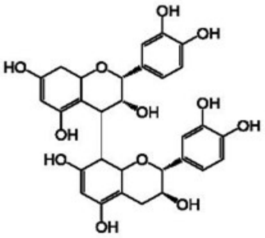
Epigallocatechin	970-74-1	306.07	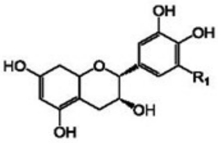
Gallocatechin	970-73-0	306.07	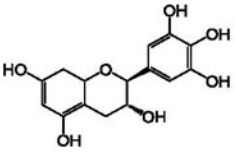

Figure S9 Information of bioassay of different forms of tannins to TSSM. (**A**) The bioassay of tannins to TSSM was performed using the soaking method. The food dyes were used to visualize the absorption of the tested chemical solution (Indicated by the red arrow). (**B**) Information of tested chemical.

Table S1 Primers used for different purposes in this study

Purpuse	Primer name	Sequence (5'-3')
**RT-qPCR**	qPCR-*MeF3H*-F	TATCCATAGCCACATTCCAG
	qPCR-*MeF3H*-R	TCCTTGCTCATCTTCCTCCT
	qPCR-*MeDFR*-F	GGATGGATGTATTTCGTGTC
	qPCR-*MeDFR*-R	AGGCTTGGTGGCATAGATGG
	qPCR-*MeANS*-F	ATGGGTGACAGCCAAATGCG
	qPCR-*MeANS*-R	AGCAAATGTGCGAGGAGGGT
	qPCR-*MeLAR*-F	GATCCAGTGGAGCCAGGTCTT
	qPCR-*MeLAR*-R	CAATGTCTGTCCCAGCAACGA
	qPCR-*MeANR*-F	AGTCAGGGACCCAGATAACCA
	qPCR-*MeANR*-R	GCTGGCTTGATCATGTCATTCT
	qPCR-*Meactin*-F	TGATGAGTCTGGTCCATCCA
	qPCR-*Meactin*-R	CCTCCTACGACCCAATCTCA
**CDS clone**	CDS -*MeLAR*-F	GTAGGCTCTTTCGTTCGGTTC
	CDS - *MeLAR*-F	CAAATAGGGTACTGGCCACAA
	CDS -*MeANR*-F	AAAAGCCCATCCATCACAAA
	CDS -*MeANR*-R	GCCAAGCAAGATCATTCATACAA
**Plasmid construct**	PC-*MeLAR-BamHI*	CGGATCCGATGACTGTTCCTGCAG
	PC-MeLAR-*XbaI*	CTCTAGAGGATAAATCATTGGCAATCCA
	PC-*MeANR-BamHI*	CGGATCCGATGAATCCCAGAAACC
	PC-*MeANR-XbaI*	CTCTAGAGACACTTCAATTCAGCAGTCC
**Southern blot**	Probe-HPT-F	CTGAACTCACCGCGACGTCTGTC
	Probe-HPT-R	TAGCGCGTCTGCTGCTCCATACA
**Positive screening**	PS-*MeLAR*-F	ATGGCCCTCCATTATTTACACT
	PS-*MeLAR*-R	CAAATAGGGTACTGGCCACAA
	PS-*MeANR*-F	TGTGAAGATAGTGGAAAAGGAAGG
	PS-*MeANR*-R	GCCAAGCAAGATCATTCATACAA
